# Patient reported psychosocial functioning following successful ptosis surgery

**DOI:** 10.1038/s41433-021-01685-w

**Published:** 2021-07-29

**Authors:** H. S. Richards, E. Jenkinson, P. White, R. A. Harrad

**Affiliations:** 1 Centre for Appearance Research, University of the West of England, Bristol, UK; 2 Bristol Eye Hospital, Bristol, UK

**Keywords:** Quality of life, Health services

## Abstract

**Background:**

Ptosis may result in increased anxiety, appearance-related distress and social avoidance, and impacts visual function. Previous work demonstrates the benefits of ptosis surgery for health-related quality of life, but there is a paucity of research comparing such outcomes before and after surgery. The aim of this study was to determine potential patient benefits in health-related quality of life, social dysfunction and anxiety following successful ptosis surgery using validated measures.

**Methods:**

Adult ptosis correction surgery patients completed validated measures of appearance-related social anxiety and avoidance, anxiety and depression, and fear of negative evaluation pre-surgery. Following successful surgery, these measures were repeated post-discharge in addition to another health-related quality of life measure.

**Results:**

Of 61 patients recruited, follow-up measures were sent to 33 and completed by 23. Paired samples t-tests demonstrated positive significant changes in appearance-related social distress pre-op *m* = 30.94, post-op *m* = 23.67 (t(17) = 3.46, 95% CI 2.84–11.72, *p* = 0.003), anxiety pre-op *m* = 7.6, post-op *m* = 4.9 (t(19) = 4.27, 95% CI 1.38–4.02, *p* < 0.001) and fear of negative evaluation pre-op *m* = 34.79, post-op *m* = 31.26 (t(18) = 2.47, 95% CI 0.52–6.53, *p* = 0.024). There was no significant difference in depression scores pre-op *m* = 3.6; post-op *m* = 3.2 (t(19) = 0.672, 65% CL −0.85 to 1.65, *p* = 0.510). In total, 85% of patients reported positive benefit to well-being following surgery.

**Conclusion:**

Increasingly, evidence suggests ptosis surgery may benefit patient’s well-being, appearance-related social anxiety and avoidance, as well as improving visual function. These psychosocial benefits should be considered alongside functional benefits in the provision of ptosis surgery.

## Introduction

Ptosis is the drooping of one or both eyelids which may result in visual impairments and altered facial appearance, and may be congenital or acquired. The global prevalence of ptosis has not been established, but incidence increases with age, with a UK study finding up to 11.5% of elderly adults are affected [1]. This aponeurotic (e.g. age-related) ptosis is the most common cause of acquired ptosis [2], often resulting from thinning or weakening of the levator muscle. Recent research has suggested that screening for ptosis should occur as standard for patients over the age of 66 [3]. Visual implications of ptosis include blurred vision, decreased visual fields and reduced ability to conduct daily activities [4]. There is relatively little research around the appearance aspects of ptosis, despite patients with a variety of ophthalmic conditions including ptosis, thyroid eye disease and strabismus reporting clinical depression, anxiety and high levels of appearance-related distress and social avoidance [5] compared to population norms [6, 7]. Individuals with appearance altering eye conditions are viewed by others as less attractive, less likeable and less successful than their peers and/or people without eye conditions [8, 9]. Patients who have undergone successful ptosis correction surgery report positive impacts on psychosocial function and well-being in post-operative interviews [10], and blepharoplasty has been shown to improve patient satisfaction and reduction in psychosocial distress related to appearance concerns [11]. However, within the NHS there are widespread inconsistencies regarding access to oculoplastic surgeries, with some Clinical Commissioning Groups (CCGs) categorising ptosis repair as a ‘cosmetic’ procedure [10]. With funding restraints often resulting in many such procedures being deemed non-essential, evidence of clinical and cost effectiveness are required to justify providing patients with these services [12].

Surgical treatments for ptosis tend to have high clinical success rates ranging from 78 to 97% [13, 14]. Although the psychosocial impact of ptosis is recognised as an important factor in the treatment of congenital ptosis in children [15, 16], patient reported outcomes relating to psychosocial function in adults are not routinely collected and rarely reported in research, with most outcome measures relating to objectively measured eyelid position [17–19]. However, there may be inter and intra-observer variability in the measurement of eyelid positions [20], and discrepancies between objective and subjective assessment of ptosis surgery outcomes [19]. The importance of patient reported outcome measures in healthcare is widely recognised [21, 22]. Where studies of ptosis have measured patient reported outcomes, these tend to focus on changes to vision and related functional quality of life [18, 23, 24]. However, studies focusing on changes to health-related quality of life (HRQL) following ptosis surgery indicate long-term benefits [12, 25], and recent qualitative work suggests a clear psychosocial benefit of successful ptosis treatment for some patients [10]. There is a paucity of studies employing repeated measures before and after corrective surgery.

The aim of this study was to measure patient benefits in HRQL, social dysfunction and anxiety relating to the impact of the appearance of ptosis following successful ptosis surgery. Validated measures were used to investigate the differences between pre and post intervention scores on constructs explored in previous qualitative and quantitative research [5–7, 10, 12, 25].

## Methods

Between September 2008 and September 2012 adult patients attending the Bristol Eye Hospital were invited to participate, with last patient in follow-up until September 2014. Patients were eligible if they were over 18 years of age, were fluent in English, presented with unilateral or bilateral ptosis of any aetiology and had been listed for surgery. Patients were included regardless of additional conditions or visual acuity and completed baseline measures at clinic visits prior to surgery. Of these, patients who had undergone clinically successful ptosis correction surgery were approached by post by a researcher (HSR) to complete follow-up measures after they had attended their final discharge appointment. Clinically successful ptosis was defined based on margin-reflex distance. The post-operative time period varied depending on number of post-operative appointments attended prior to discharge from care and appointment attendance rates.

### Measures

Three validated psychological measures were administered to all participants prior to their ptosis surgery (outlined below). Participants who had undergone clinically successful ptosis correction surgery were asked to complete the measures again, with an additional post-operative measure, following their discharge from post-surgery clinic. As follow-up measures were not completed until after patients had attended discharge appointment, the length of time from surgery to post-operative questionnaire completion differed between patients.

- The Derriford Appearance Scale – Short Form (DAS24) is a 24-item measure of distress and loss of function due to perceived problems with appearance. Scores range from 11 to 96 with higher scores indicating poorer adjustment. The scale demonstrates good internal reliability (Cronbach’s alpha = 0.92). DAS24 scores >30 are considered indicators of higher levels of appearance-related anxiety impacting on quality of life compared to the general population [26], with scores over 47 indicating considerable levels of appearance-related distress and social avoidance [5]. The DAS24 demonstrates good six-month test-retest reliability in a clinical population (*r* = 0.82), and good three-month test-retest reliability in a general population (*r* = 0.88) [26].
- The Hospital Anxiety and Depression Scale (HADS) is a 14 item measure of acute levels of depression (7 items) and anxiety (7 items). Scores range from 0 to 21 for each scale with increasingly higher scores indicating increasingly greater levels of distress. Scores can be categorised as possible or probable cases of depression and anxiety. A score on either of the anxiety or depression subscales below 7 indicate no clinical anxiety or depression. A score of 8–10 indicates moderate levels of anxiety or depression, and a score of ≥ 11 is indicative of clinical anxiety or depression. The HADS demonstrates good internal consistency and good concurrent validity in comparison to other similar measures (range of *r* = 0.60–0.80) and shows good test-retest reliability (*r* = 0.70–0.80) [27].
- The brief version of the Fear of Negative Evaluation scale is a 12-item measure of apprehension about the evaluations of an individual made by others and the distress caused by these perceived evaluations. The FNE demonstrates good internal consistency (*a* = 0.97) and high test-retest reliability (*r* = 0.94) [28]. Higher scores indicate higher levels of distress, with score >25 considered to be an indicator of social anxiety [29].
- The Glasgow Benefit Inventory is used to determine patient benefit following an intervention, defined as the patient’s perspective of psychological, social and physical wellbeing. It is delivered post intervention to measure the changes in health status in three subscales – general, social support and physical health. Scores are calculated to produce a result ranging from +100 (maximal positive benefit) to −100 (maximal negative benefit) with 0 indicating no change in health status.

### Data analysis

It was anticipated pre-study that successful surgery would have a meaningful positive impact on appearance anxiety, anxiety of perceptions of negative evaluation. Informed by our previous work [6, 7, 10, 30] we estimated the standardised effect size (Cohen’s d) for pre- post change to conservatively be 0.6 as the population is one of successful surgery. We used the proprietary software PASS (Power and Sample Size) for the power calculation and sample size determination and confirmed using Minitab. A sample size of *n* = 23 complete pre- and post- evaluations would have at least 80% power to detect a repeated measures standardised effect size of 0.6 or larger using the paired samples t-test (alpha = 0.05, two-sided).

Two pass data entry was undertaken to ensure coding fidelity and data veracity. Data validity checks were undertaken and derived scale data was examined for the presence of any unduly inferential observations. Non-responders at follow-up did not appreciably differ from follow-up responders, with high item completion rates among responders consistent with data missing completely at random (MCAR). For these reasons, the analyses were conducted on an available case basis [31]. For scale data, an assessment of change in psychosocial functioning following successful operation was undertaken using the paired samples *t*-test, with 95% confidence intervals (95% CI) for mean difference, and Cohen’s *d* for repeated measures data to quantify effect size. The percentage of patients transitioning between clinical thresholds are reported to additionally quantify effect on psychosocial functioning.

### Statement of ethics

We certify that all applicable institutional and research governance guidance concerning the ethical use of human volunteers were followed during this research. Ethical approval was gained from the South West Central Bristol Research Ethics Committee (09/H0106/51).

## Results

Seventy-eight patients were approached, and 61 patients provided informed consent to take part and completed pre-operative measures [7]. For all pre-op ptosis patients, the margin-reflex distance was 1 mm or less. Ptosis surgery was determined to be successful when patients and the doctor were happy with the outcome, the margin-reflex distance was never less than 2 mm and the difference in margin-reflex distance between the two eyes was never more than 1 mm. Follow-up measures were sent by post to participants whose surgery was deemed to be successful after they had completed their final discharge appointment at Bristol Eye Hospital (*N* = 33). Two participants who had successful ptosis surgery did not attend their final discharge appointment. Ten participants were lost to follow-up during a six-month period due to staffing issues and were not sent follow-up measures. 23 participants returned completed post-operative follow-up measures, giving a response rate of 70%, at which point follow-up measures ceased to be sent out. The average time since surgery upon completion of follow-up measures was 14 months (range 4–30 months). Independent t-tests demonstrated that there were no significant differences in the pre-operative scores of participants who did and did not return their follow-up measures in terms of appearance concern (returners *m* = 31.41, SD11.48, non-returners *m* = 30.5, SD 13.19, t(30) = 0.198, *p* > 0.05, 95% CI −8.45 to 10.27), anxiety (returners *m* = 7.6, SD = 4.94, non-returners *m* = 6.25, SD = 3.88, t(26) = 0.689, *p* > 0.05, 95% CI −2.67 to 5.37), depression (returners *m* = 3.6, SD = 3.13, non-returners *m* = 3.75, SD = 3.05, t(26) = −0.115, *p* > 0.05, 95% CI −2.82 to 2.52) and fear of negative evaluation (returners *m* = 34.5, SD = 10.59, non-returners *m* = 30.5, SD = 12.17, t(26) = 0.877, *p* > .05, 95% CI −5.44 to 13.54).Participant demographics are summarised in Table 1.

**Table 1:** Participant demographics.

Demographics	Responders *N* (%)	Non-responders *N* (%)
Total	23 (70)	10 (30)
Sex		
Male	9 (39)	5 (50)
Female	14 (61)	5 (50)
Age		
Mean	66	55
Range	28–89	18–73
Ethnicity (self-described)		
White	20 (87)	5 (50)
Did not disclose	3 (13)	3 (30)
Black	0	1 (10)
Indian	0	1 (10)
Relationship status		
Single	2 (9)	3 (30)
Married	15 (65)	6 (60)
Divorced/Widowed	2 (9)	0
Did not disclose	4 (17)	1 (10)

All patients presented with aponeurotic ptosis. Seven patients (30%) underwent bilateral simultaneous ptosis correction surgery and the remaining 16 (70%) underwent surgery for right (*n* = 8) or left (*n* = 8) eye only. All procedures were considered clinically successful by a consultant.

Twenty-two (96%) and eighteen (78%) patients completed the DAS24 pre- and post-surgery respectively. Twenty (87%) and twenty-one (91%) patients completed the HADS pre- and post-surgery respectively. Twenty (87%) patients completed the FNE at both time-points. Twenty-one patients (91%) completed the GBI questionnaire.

A paired samples *t*-test was conducted to compare DAS24 scores before and after successful ptosis surgery. There was a significant difference in the scores, with post-surgery scores (*M* = 23.7, SD = 7.9) being lower than pre-surgery (*M* = 30.94, SD = 10.9); t(17) = 3.46, 95% CI 2.8–11.7, *p* = 0.003 with a medium effect size (*d* = 0.66).

A paired samples *t*-test was conducted to compare levels of depression and anxiety as measured by HADS pre and post successful surgery. There was a significant difference in anxiety scores, with post-surgery (*M* = 4.9, SD = 4.1) scores being lower than pre-surgery (*M* = 7.6, SD = 4.9); t(19) = 4.27, 95% CI 1.38–4.02, *p* < 0.001 with a medium effect size (*d* = 0.55). However, there was no significant difference between depression scores before (*M* = 3.6, SD = 3.1) and after successful ptosis surgery (*M* = 3.2, SD = 4.4) (t(19) = 0.672, 65% CL −0.85 to 1.65, *p* = 0.510).

A paired samples t-test was conducted to compare levels of fear of negative evaluation pre and post successful surgery. There was a small significant difference, with post-operative (*M* = 31.3, SD = 11.8) scores being lower than pre-operative (*M* = 34.8, SD = 10.8); t(18) = 2.47, 95% CI 0.5–6.5, *p* = 0.024 with a moderate effect size (*d* = 0.32). All participants either retained the same outcome or improved on each and every measure apart from two patients, one who went from “normal” to “possible” on HAD Depression and another who went from “possible” to “probable” on HAD Depression. This is illustrated in Table 2.

**Table 2:** Summary of changes to patients scoring within thresholds of DAS24, HADs and FNE pre and post-surgery.

Pre-Intervention		Post-Intervention		
		Derriford appearance scale post intervention		
		Minimal Concern	High Concern	
Derriford Appearance Scale Pre Intervention	Minimal concern^a^	9	0	
	High concern	5	4	

		Fear of negative evaluation post intervention		
		No Social Anxiety	Social Anxiety	
Fear of Negative Evaluation Pre Intervention	No Social Anxiety	4	0	
	Social Anxiety	4	11	

		HAD anxiety post intervention		
		No Clinical Anxiety	Moderate Anxiety	Clinical Anxiety
HAD Anxiety Pre intervention	No Clinical Anxiety	11	0	0
	Moderate Anxiety	1	1	0
	Clinical Anxiety	2	2	3

		HAD Depression Post Intervention		
		No Clinical Depression	Moderate Depression	Clinical Depression
HAD Depression Pre Intervention	No Clinical Depression	16	1	0
	Moderate Depression	0	2	1
	Clinical Depression	0	0	2

^a^Score thresholds are provided in Measures section.

Post-surgery, 78% of participants reported minimal levels in appearance-related anxiety compared with 41% pre-surgery, and post-surgery there were no reports of considerable appearance anxiety compared with 14% pre-surgery, as seen in Table 2. Similarly, benefits in anxiety were observed with the percentage reporting clinical anxiety decreasing (35% pre-surgery, 14% post-surgery) and with those reporting no indication of clinical anxiety improving (55% pre-surgery, 72% post-surgery).

The Total GBI score average was 20.5 (SD = 16.1, range −6 to +58, 95% CI 13.2–27.8 *p* < 0.05), indicating an overall positive benefit on wellbeing. However, the large standard deviation indicates a wide range of responses. In accordance with Hendry et al. [32], we report percentage of patients reporting negative change, no change and positive change to health status following intervention. 5% reported negative change following successful ptosis surgery, 10% reported no change following successful ptosis surgery and 85% reported positive change following successful ptosis surgery on the General subscale. GBI total and subscale scores are presented in Table 3.

**Table 3:** Summary of GBI total and subscale scores.

GBI subscale	Mean (SD)	Range	% negative change	% no change	% positive change
Total score (*n* = 21)	20.5 (16.1)	−6 to 58	10%	5%	85%
General	30.9 (24.2)	−8 to 88	5%	10%	85%
Social	3.2 (8.5)	0 to 33	0%	85%	15%
Physical	−3.9 (17.4)	−67 to 17	14%	76%	10%

## Discussion

This study examined levels of psychosocial function related to appearance and social anxiety before and after clinically successful ptosis surgery using validated measures. Results indicate that post surgery, patients report lower levels of distress relating to appearance concerns, lower levels of general anxiety and reduced concern about negative evaluation from others. The majority of patients reported positive changes as a result of treatment, with GBI scores indicating that ptosis surgery is of benefit to patients in improving self-reported psychosocial well-being related to appearance as measured by the DAS24, HADS, FNE. There was no significant difference found between pre and post-operative reported levels of depression. Although levels of clinical depression reported in this cohort were generally low, this lack of a significant change may suggest that distress relating to ptosis symptoms is linked more to anxiety rather than depression. Similar trends indicating higher levels of anxiety compared to depression have been found in other ptosis [6], strabismus [30] and ophthalmic [5] patient groups.

Comparison of pre and post-operative scores demonstrate that following clinically successful ptosis surgery, no patients reported considerable levels of appearance anxiety and distress impacting on social function. Fewer patients reported higher levels of appearance concern, with the majority reporting levels consistent with non-clinical populations [26]. Similar findings were demonstrated for anxiety and distress relating to perceived judgement from others, with approximately a quarter and a fifth of patients reporting substantial improvements respectively. The benefits of ptosis surgery were further illustrated by 85% of patients reporting a positive change in health status, with these GBI scores similar to other post-operative ptosis patient groups [12, 25, 33]. Although 14% of patients reported a negative change in physical health status, since the GBI covers a range of potential health problems this may reflect the fact that many in this patient group are elderly; there are also similar findings in other studies [12, 25, 33]. Although there were minimal benefits reported in the GBI social subscale, it is worth noting that these questionnaire items relate to perceived levels of social support received/required following treatment, rather than changes in social function and interactions with others.

This study has some limitations which should be considered when interpreting results. Patients were not recruited consecutively, were from a single site and represent a relatively small sample. Follow-up measures were only sent to patients who completed all of their follow-up consultations and whose surgery was deemed to be clinically successful. As such, patients who disengaged from treatment or did not have outcomes deemed to be objectively successful were not included in follow-up, and these factors may limit the generalisability of findings. However, results presented here are similar to those found in other ptosis patient groups [6, 12, 25, 33]. Time between surgery and completion of follow-up measures varied between patients from 4 to 30 months. However, ptosis surgery demonstrates a sustained patient benefit over time [12], with measurable perceived benefits present at 32 months post-surgery and no significant differences in benefits between short-term and long-term patient follow-up groups [25].

The current findings add to a growing body of evidence indicating that ptosis surgery is of substantial benefit to patients [10, 12, 25, 33]. Previous research tends to be retrospective [12, 25, 33] or has focused predominantly on functional improvements [17–19, 34]. To our knowledge, this is the first prospective study to examine changes in psychosocial wellbeing and function in blepharoptosis patients. By demonstrating improvements to HRQL and psychosocial function, these findings should inform the provision of ptosis surgery services, as patient benefit is not limited to improving visual impairments but instead may include wider psychological, functional and social benefits.

### Summary table

#### What was known before

- Ptosis detrimentally impacts vision, and previous research has demonstrated a negative impact on psychosocial function.
- This can include social avoidance and anxiety.
- Access to treatment is inconsistent throughout the NHS, and is largely based on visual function.
- Patient reported outcomes are rarely collected or analysed in this patient population.
- There is a paucity of repeated measures studies examining psychosocial impact in ptosis patients, although qualitative evidence demonstrates clear psychosocial benefits following successful surgery.

#### What this study adds

- The results of this study show positive significant changes for adult patients with ptosis in appearance-related social distress, anxiety and fear of negative evaluation from others following successful surgery which has not been demonstrated in previous literature.
- These psychosocial benefits should be considered alongside functional benefits in the future research and in clinical practice.
